# Beads in the Tooth

**DOI:** 10.5005/jp-journals-10005-1103

**Published:** 2010-04-15

**Authors:** Jyothsna V Setty, Ila Srinivasan

**Affiliations:** 1 Professor, Department of Pedodontics and Preventive Dentistry, KLE Society’s Institute of Dental Sciences Bengaluru, Karnataka, India; 2 Professor and Head, Department of Pedodontics and Preventive Dentistry, KLE Society’s Institute of Dental Sciences Bengaluru, Karnataka, India

**Keywords:** Foreign objects, Beads, Retrieval, Simple procedure.

## Abstract

Foreign objects in a tooth are often diagnosed accidentally. A detailed case history, clinical and radiographic examinations are necessary to know the exact nature, size, location of the foreign body and the difficulty involved in its retrieval. In the present case, two beads, one radiopaque and one radiolucent were found in the same tooth at different places of 11-year-old girl. Patient did not reveal proper history out of fear. Both the foreign objects were discovered during routine endodontic procedure which were removed following simple clinical procedure causing minimal damage to the internal tooth structure.

## INTRODUCTION

There have been several reports describing the placement, by patients, of foreign objects into the exposed pulp chambers and canals. Foreign objects inserted in the canal have varied from radiolucent objects, like wooden tooth picks, broomstick or toothbrush bristle to radiopaque materials like paper pins, needles, screws, beads and pencil leads.^1-3^ The discovery of such foreign bodies in the teeth is a special situation, which very often is diagnosed accidentally. More so, if such situation is not revealed by patient, which very often is common among children. It is a well-known fact that children often tend to have the habit of placing foreign objects in the mouth and these objects can get lodged in the pulp chambers and root canals of the teeth. These impacted objects often act as potent source of infection. Retrieval of such foreign objects from the tooth of the child is a challenging aspect in pediatric dental practice. These objects can be easily retrieved if they are located within the pulp chamber, but if the object is pushed more apically into the root canal, the retrieval of such objects may be a complicated procedure especially if the object is radiolucent and not diagnosed by diagnostic radiographs.

The following is a case report describing the retrieval of foreign objects from a tooth of a child patient and its successful management.

## CASE REPORT

An 11-year-old girl presented to the Department of Pedo-dontics and Preventive Dentistry, KLE Society’s Dental College, Bengaluru, India, with the chief complaint of pain in the upper anterior region since 6 days and swelling since 2 days. Patient gave the history of trauma 8 months back for which the patient had undergone treatment with the local dentist and the tooth was asymptomatic till the recent episode of pain 6 days back. Patient’s parents also said that they visited the same dentist 5 days back for the pain and the dentist offered extraction of that particular tooth as the only treatment option. Parents were unwilling for extraction, so they visited our hospital for second opinion.

**Fig. 1:**
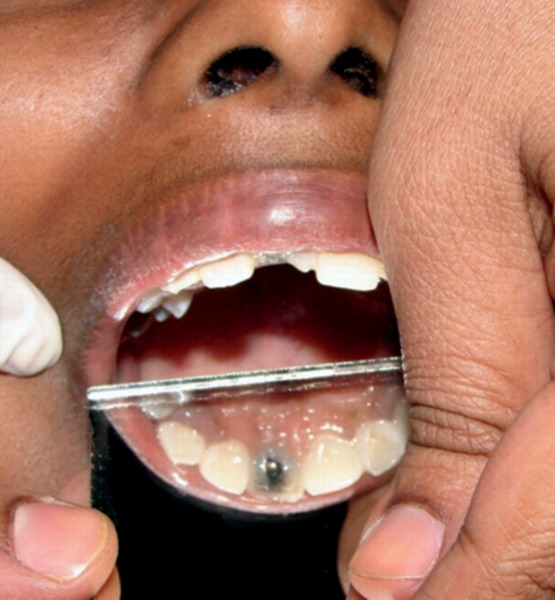
Fractured right maxillary central incisor tooth with the foreign object

**Fig. 2:**
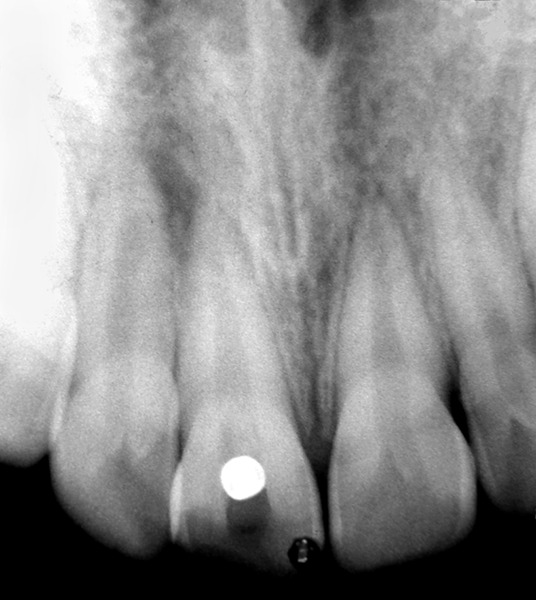
Preoperative radiograph showing the fractured right maxillary central incisor with a radiopaque foreign object

On examination right central incisor was fractured, discolored and tender on percussion with the palatal surface showing access cavity preparation, filled with food debris. Radiographic examination revealed circumscribed radiopacity at the canal orifice extending into the pulp chamber (Fig. 1). The tooth was isolated, access cavity was cleaned, reshaped, and the pulp chamber was irrigated with normal saline. A metallic object snuggly fitting at the root canal orifice and extending into the pulp chamber was seen (Fig. 2). A thin tapering fissure bur was used to slightly widen the orifice of the canal, and ultrasonic scaler was used to clear the debris. The circular object started rotating slightly in place when tried to displace with the explorer and was successfully removed as it got struck to the explorer tip. The dislodged object was a metal bead. On further repeated inquiry, patient revealed that she had bitten on the bead necklace which she had worn, during which bead had entered the tooth and she was scared of consequences and feared to tell the truth.

**Fig. 3:**
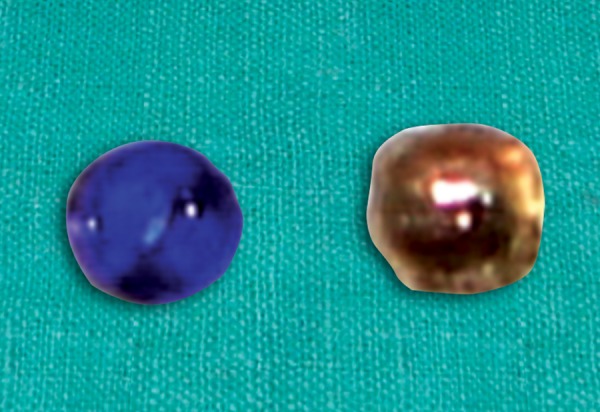
Beads retrieved from the tooth

**Fig. 4:**
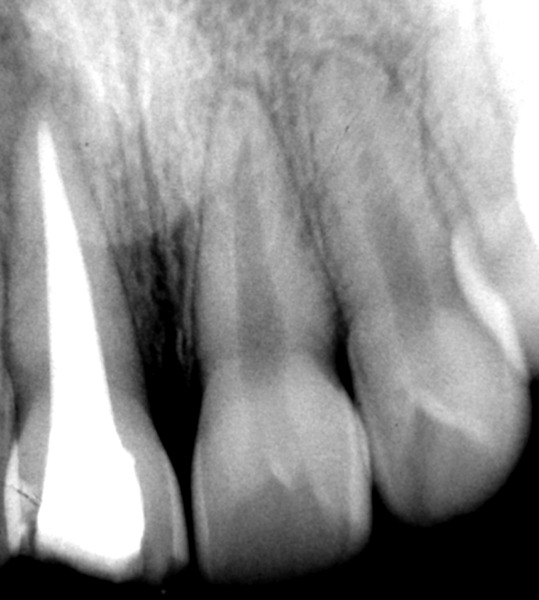
Radiograph after completion of root canal treatment

**Fig. 5:**
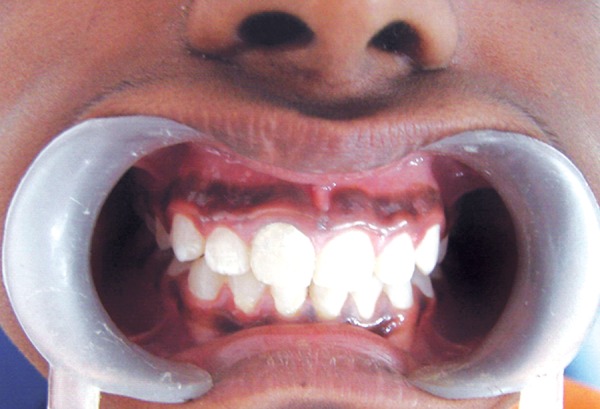
The tooth after successful restoration

With the intention of carrying out routine endodontic procedure, a no. 15 K-file was inserted into the root canal. There was a definite obstruction at 12 mm of working length. The radiograph failed to reveal any radiopaque obstruction. With the history we learnt that the obstruction might be other bead/beads which are radiolucent, as the patient’s necklace was plastic bead necklace with few metal beads in it. No. 8 K-file was used to bypass the foreign object on its sides. K-files no. 10 and no. 15 were then used to bypass the bead. Finally, two Hedstrom files no. 20 were inserted along the foreign object on its mesial and distal sides and files were pulled together. This procedure was repeated for three to four times to loosen the bead that finally came out along with the Hedstrom files. After this plastic bead (Fig. 3) was removed, the canal was negotiated to the complete working length. Routine root canal treatment was eventually completed after symptoms subsided (Fig. 4) and the crown was restored (Fig. 5).

## DISCUSSION

Removal of foreign objects from the root canal is often a tedious and difficult procedure. The procedure becomes more difficult if the foreign body is unusual and radiolucent. A radiograph can be of diagnostic significance especially if the foreign body is radiopaque. McAuliffe ^4^ summarized various radiographic methods to be followed to localize radiopaque foreign object as Parallax views, vertex occlusal views, triangulation techniques, stereo radiography and tomography. Specialized radiographic techniques, such as radiovisiography, 3D CAT scans can play a pivotal role in localization of these foreign objects inside the root canal.

There are many techniques for the retrieval of foreign objects lying in pulp chamber and root canal. Ultrasonic instruments,^3,5^ the Masserann Kit^6^ and modified Castroviejo needle holders^7^ can be used to remove the objects from the pulp chamber and root canal. A simple device consisting of a disposable 25 guage needle, a segment of a thin steel wire and a small mosquito hemostat to remove silver cone from root canal was explained by Roig-Greene.^8^ Steglitz forceps have been described for the removal of sliver points from root canal. Meidinger^5^ used Cavio-Endo ultrasonic instruments to remove foreign objects from the canal space. Srivastava and Vineeta^9^ have suggested periapical surgery or intentional reimplantation to remove foreign objects. Nehme^10^ had recommended the use of operating microscope to remove the lodged object. Removal of small amount of tooth structure to gain access to the foreign object was suggested by McCullock.^11^ Walvekar^12^ et al recommended the loosening of snuggly bound foreign body to prevent the perforation of the root.

## SUMMARY

In the above case report, retrieval of foreign objects was accomplished by using simple procedure. The first object was radiopaque and was lodged at the canal orifice and was successfully dislodged using a thin tapered fissure bur, ultrasonic scaler and explorer tip under copious irrigation. The second object was radiolucent and was snuggly fitted into the canal space which was loosened first with K files and finally removed with Hedstrom-file with minimal removal of internal tooth structure.
